# Assessment of biotoxicity of three types of landfilled foundry waste on the basis of dehydrogenase activity

**DOI:** 10.1007/s10661-022-10320-4

**Published:** 2022-08-16

**Authors:** Marta Bożym

**Affiliations:** Opole University of Technology, Prószkowska 76 Street, 45-758 Opole, Poland

**Keywords:** Foundry waste, Dehydrogenase activity (DHA), Heavy metals (HM), Spent foundry sand (SFS), Pot experiment

## Abstract

The microbiological activity of three types of landfilled foundry wastes, i.e. biologically reclaimed foundry waste (BFW), foundry waste landfilled since the 1990s (LFW) and fresh foundry waste (FFW), was investigated. The wastes originated from a Polish iron and steel foundry which uses organic binders based on phenol–formaldehyde resins and mineral binders to casting production. The physical and chemical properties and dehydrogenase activity (DHA) were determined in the waste samples and local soils. In addition, a pot experiment was performed to determine the effect of the addition of FFW with no microbial activity on soils. Additional correlation analysis was conducted between DHA and other parameters. It was found that biologically reclaimed foundry waste (BFW) showed the highest microbial activity, similar to soils from garden allotments and agricultural fields. The DHA in LFW was about a half lower than BFW. On the other hand, FFW did not show any microbial activity. A pot experiment showed that increasing the percentage of foundry waste relative to soil had a negative effect on DHA, probably as a result of soil dilution rather than the inhibitory effect of contaminants. It was concluded that the optimum addition of FFW to soils is 10% wt, due to the highest value of DHA among the other variants.

## Introduction

The annual production of the foundry industry in the world is about 105 million tons. It is estimated that each ton of castings produced results in 0.6 tons of foundry waste (Holtzer et al., 2016). The largest producer of castings is China (49 million tons). India is second with 12 million tons, followed by the USA with 10 million tons (Sabour et al., 2021). Foundry waste consists mainly of spent foundry sands (SFS) (Bożym, 2018a, 2019, 2020, 2021). Sand used for foundry molds has high physical and mechanical properties and is therefore increasingly used in construction and road building as an aggregate (Torress et al., 2017). Due to limited sand resources and environmental concerns, SFS can be used for cement production (Sabour et al., 2021). In some countries, SFS are used in agriculture, for the production of artificial soil substrates (Bożym, 2021). However, only SFS with mineral binders so-called Green sands from iron and steel foundries are suitable for this purpose, not from non-ferrous metal foundries, due to the high leachability of heavy metals (HM) (EPA Report, 2014). Foundry wastes containing organic binders are an environmental hazard due to the presence of toxic organic substances such as phenol and formaldehyde (Bożym, 2020; Dungan et al., 2009; Holtzer et al., 2016). Some toxic substances such as phenol, formaldehyde, furan, phenol–urethane, urea resins and furfuryl alcohol may be leached from landfilled metallurgical foundry waste (Bożym, 2020; Bożym et al., 2021).

In Poland, foundry waste is increasingly being recovered, both from current production and landfilled waste (Bożym, 2018a, 2019, 2020, 2021). Inactive foundry waste landfills are usually biologically reclaimed (Bożym, 2018b). Plant species that are resistant to harmful substances are usually used for reclamation, such as HM (Kicińska & Gruszecka-Kosowska, 2016). Their function is to protect the landfill from dust emission, to strengthen the slopes mechanically and to create a soil layer. Phytostabilization has the advantage of reducing erosion and creating a biologically active soil structure in the upper layer of the landfill (Remon et al., 2005). During landfill reclamation, organic fertilizers are used as a source of carbon and humus, i.e. sewage sludge (Kicińska et al., 2018). Biological reclamation also has the advantage of having a positive effect on the local landscape (Bożym, 2018b). An important aspect is to conduct continuous monitoring of soils or groundwater to assess the impact of landfills on the local environment.

Among the commonly used soil enzymatic activity tests, dehydrogenase activity (DHA) is considered to be the most useful. Dehydrogenases are intracellular enzymes, and their activity is a measure of the number of active cells in the soil (Zamulina et al., 2021). Dehydrogenases play an important role in organic matter metabolism, nutrient circulation and improvement of soil structure. DHA is an indicator of soil health and is correlated with the number and diversity of soil microorganisms (Maurya et al., 2020). Soil DHA is usually analysed using two colorimetric methods: with 2,3,5-triphenyltetrazolium chloride (TTC) (Ciarkowska & Gambus, 2020; Hamidović et al., 2020; Lemanowicz et al., 2020; Uzarowicz et al., 2020) or with iodonitrotetrazolium formazan (INTF) (El Gouzi et al., 2015; Emmert et al., 2021; Hanajík et al., 2017; Ramirez et al., 2021). Poorer results for the TTC method have been reported due to the effects of drying, temperature, moisture and oxygen in the soil (Kumar et al., 2013). The results obtained from both methods vary significantly, and so should not be compared. Dungan et al. (2006) and Zhang et al. (2014) proposed the INTF method for the analysis of SFS used in agrotechnics. For this reason, this method is also used in the current study. Until now, no DHA tests in foundry waste have been performed. Due to the possibility of using SFS in agriculture and horticulture (EPA Report, 2014), the results of dehydrogenase activity as one step of the evaluation of their biotoxicity and the possibility of the reuse of these SFS in agrotechnics are presented in the current study.

Currently, SFS toxicity studies are mainly based on the leachability of contaminants (Bożym, 2017, 2019, 2020; EPA Report, 2014). The biotoxicity of SFS is also assessed in pot, plot, and vegetation experiments (Lindsay & Logan, 2005). SFS are used in the production of so-called Technosols (EU, 2006; EPA Report, 2014; Barredo et al., 2020); these are soils developed on non-traditional substrates and anthropogenical areas (IUSS Working Group WRB, 2015). Some authors use the term Technosols for contaminated and degraded soils of industrial areas (Lemanowicz et al., 2020; Uzarowicz et al., 2020; Zamulina et al., 2021). In the study of Technosols of industrial sites, some authors used DHA analyses as an indicator of the degree of their remediation. These analyses may also be useful for the evaluation of Technosols based on SFS or reclaimed landfills. Usually, higher DHA values are found in topsoils and top layers of Technosols (Lemanowicz et al., 2020; Uzarowicz et al., 2020). DHA in Technosols is negatively correlated with pH and salinity (Filipovic et al., 2020; Uzarowicz et al., 2020) and HM (Ciarkowska & Gambus, 2020). It should be noted that HM may reduce DHA in soil only in higher concentrations (Hamidović et al., 2020; Maurya et al., 2020; Tiwari et al., 2019). The increase of DHA in soils and Technosols is influenced by organic fertilisation and plant cover (Brkljaca et al., 2019; Howard, 2017) due to the higher soluble organic matter content (Zamulina et al., 2021), while the impact of agrotechnical measures is still being investigated (Tiwari et al., 2019). In a study of biologically reclaimed landfills of foundry waste, Remon et al. (2005) found low toxicity of the top layers, called by the authors ‘soils’, due to the activity of plant roots and the high content of organic matter. Similarly, Zamulina et al. (2021) found that plant cover and the content of soluble organic matter in soil contaminated with HM are factors that increase DHA. Based on the experience of other authors, it can be assumed that the top layer of biologically reclaimed landfills of foundry waste is characterised higher a DHA value than lower layers or fresh waste.

The aim of this study was to investigate the microbial activity of landfilled foundry waste and to evaluate its toxicity. These wastes can be considered a component of Technosols or used in agrotechnics due to their low toxicity, e.g. compared to dust (Bożym, 2019, 2020, 2021, 2022). Additionally, the effect of the biological reclamation of foundry waste on microbial activity was evaluated. A pot experiment was conducted to demonstrate the effect of fresh foundry waste (FFW) on soil properties and soil microorganisms. The enzymatic activity studies were complementary to previous studies of the leachability of pollutants and evaluation of the germination index and bioaccumulation tests (Bożym, 2017, 2020, 2021, 2022). The study benefited from the experience of other authors investigating the suitability of foundry waste for agrotechnical applications.

## Material and methods

### Sampling

**Fig. 1:**
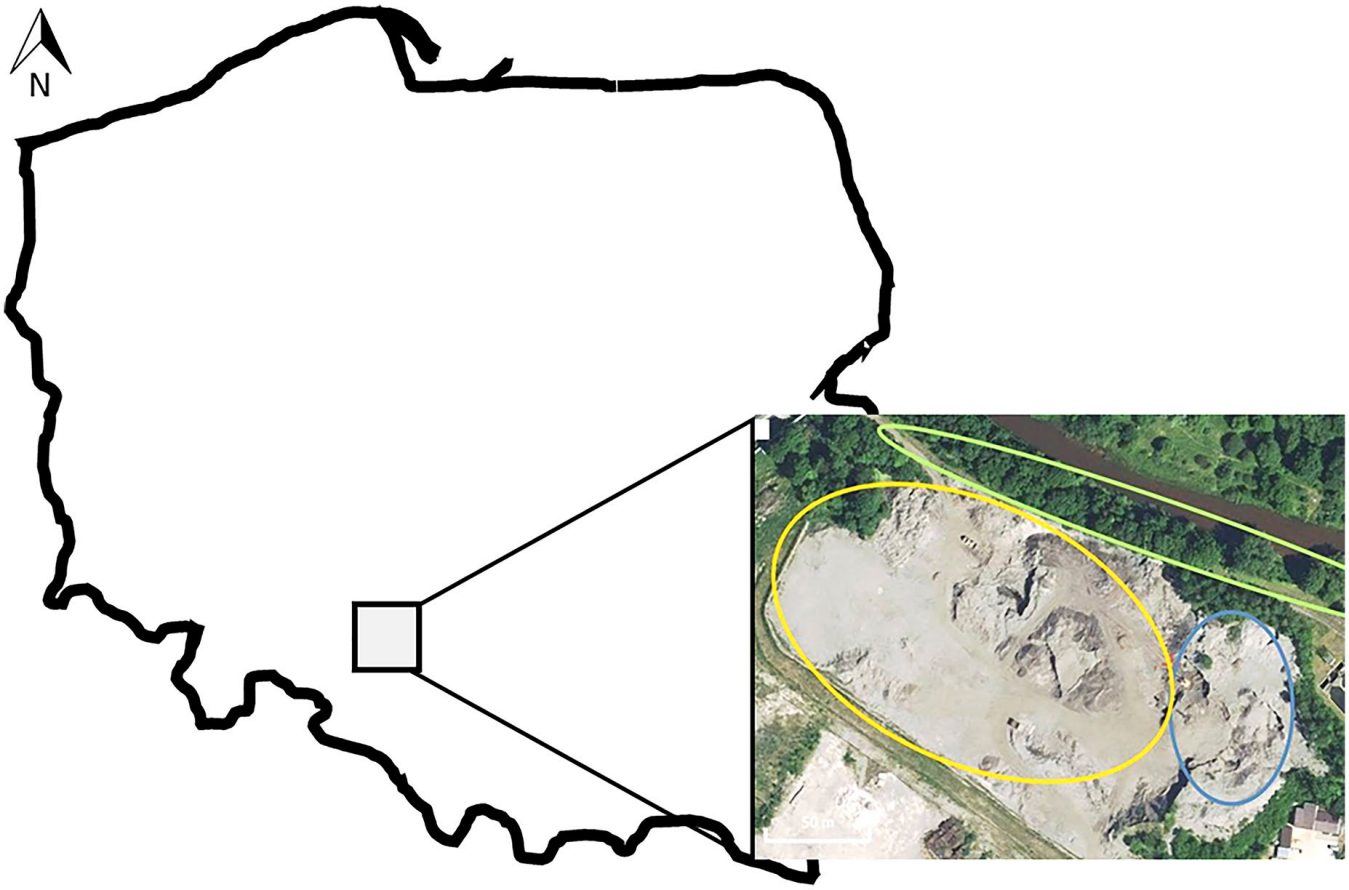
Location of foundry landfill, Opole Voivodeship, Poland (source: Google Maps)

Three types of samples of foundry waste were collected in 2017/18 from the waste landfill from one of the Polish foundries located in southern Poland (N 50.677431904906406; E 18.205493688583378) (Fig. 1). Until 2003, approximately 3.5 million tonnes of foundry waste were collected on this landfill (Bożym, 2019). Since then, waste has been recovered and reused for the production of building aggregates. The first type of taken samples were landfilled foundry waste (LFW) (*n* = 6; in yellow, Fig. 1). The collected LFWs were landfilled since the 1990s of the twentieth century. These wastes consisted mainly of used SFS with organic binders, additionally in a smaller amount refractory materials, slags and dust. Samples were collected from six piles formed by a shredder-sorting device during the recovery. The processed material (fi 4 mm) is used for the production of road aggregates of various granulation. The second type of waste was collected from five points of the current production pile located in the landfill. This waste was given the term ‘fresh foundry waste’ (FFW) (*n* = 5, in blue, Fig. 1). The FFW consisted of SFS, with no other waste added. The third group of waste was collected from the embankment of landfill area. This waste was given the term ‘biologically reclaimed foundry waste’ (BFW) (*n* = 10, in green, Fig. 1) (N 50.67858087270267–50.67777863779647; E 18.204313516616825–18.207263946533207). The embankment with a length of approx. 250 m and a height of approx. 5 m was to separate the landfill from the river, located about 10 m from the landfill (Bożym, 2018a, b). The embankment was biologically reclaimed by planting trees and grasses as well as natural succession of local plants. The samples were taken at a distance of approx. 25 m from each point at a height of about 2–3 m of the embankment and a depth of 20 cm after removing the turf. The samples were collected in accordance with the Polish soil standards (PN-ISO 10381–1-5).

**Fig. 2:**
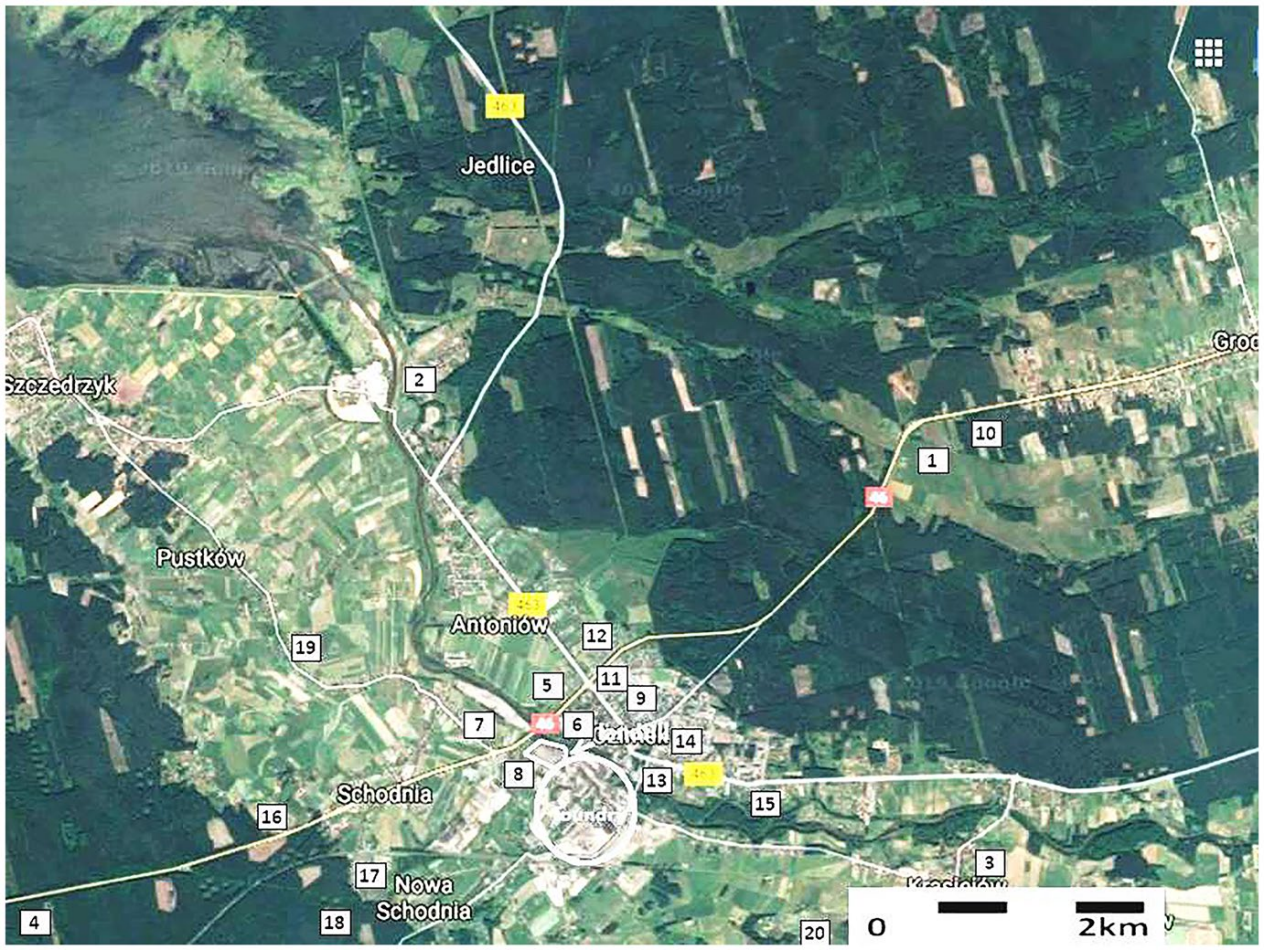
Location of soil sampling points (source: Google Maps)

In order to compare the results of microbiological activity of the landfilled waste, soil samples (*n* = 20) were collected at different distances from the foundry. The location of soil sampling sites is presented in Fig. 2. The characteristics of soil sampling points are presented in Table 1.

**Table 1:** Characteristic of soil sampling point

No	Type of sampling point	Geo-coordinates
1	Meadows	(N 50.694935316246315; E 18.24262619018555)
2	Arable land	(N 50.70102429235463; E 18.189883232116703)
3	Arable land	(N 50.669782733786846; E 18.254213333129886)
4	Arable land	(N 50.66510422644469; E 18.142933845520023)
5	Allotment gardens	(N 50.68187125206409; E 18.207306861877445)
6	Wasteland	(N 50.67919273756558; E 18.206019401550297)
7	Arable land	(N 50.67965503017147; E 18.197522163391117)
8	Arable land	(N 50.67492991322267; E 18.202993869781498)
9	Wasteland	(N 50.68057280346798; E 18.21269273757935)
10	Forest soil	(N 50.69961085050092; E 18.249492645263675)
11	Allotment gardens	(N 50.68109626612491; E 18.20842266082764)
12	Allotment gardens	(N 50.68386984094747; E 18.21067571640015)
13	Allotment gardens	(N 50.67454236453823; E 18.219559192657474)
14	Wasteland	(N 50.6775338854341; E 18.21964502334595)
15	Allotment gardens	(N 50.67499110482774; E 18.235738277435306)
16	Arable land	(N 50.6729649403849; E 18.175796270370487)
17	Arable land	(N 50.66868115047462; E 18.186535835266117)
18	Forest soil	(N 50.663771310538905; E 18.17971229553223)
19	Arable land	(N 50.68503904395654; E 18.18048477172852)
20	Arable land	(N 50.66317284619182; E 18.239192962646488)

The characteristics of the tested soils are presented in the publication by Bożym and Klojzy-Karczmarczyk (2021). The tested soils are mainly light soils (clay sands, slightly clay sands and loose sands), characteristic of the areas of south-western Poland (Ostrowska et al., 1991). The granulometric composition was analysed according to the Polish Soil Science Society and the industry standard BN-78/9180–11 in force in Poland until 2008. These guidelines are still applied in practice due to the significant achievements of Polish soil science and the use of source materials developed before 2008, including the valuation classification of agricultural land, or soil and agricultural maps. In previous studies, no contamination of local soils with HM was found, which indicated no negative impact of the foundry on the local environment (Bożym & Klojzy–Karczmarczyk, 2021). Soil samples were collected with the use of a soil stick from a depth of 20 cm from several points in the area of 100 m × 100 m with a total mass > 1 kg, in accordance with the Polish standard PN-R-04031 and PN-ISO 10381–1-5.

### Physicochemical parameters

All analyses were performed in accordance with reference methods based on Polish standards or literature. The dry matter content of the samples was determined in 105 °C (PN-EN 15,934–02); the loss on ignition (LOI) in 550 °C (PN-EN 15,169); pH in H_2_O using pH-conductometer CPC 501 (Elmetron) (PN-ISO 10390); C-org by the titration method of Tiurin according to Ostrowska et al. (1991), total nitrogen was determined by elemental analysis using a CHNS analyzer (Elementar Vario Macro Cube) (PN-ISO 13878); total phosphorus was determined by spectrophotometric method after sample mineralization (PN-EN 14,672); HM (Cd, Pb, Cu, Zn, Ni, Cr, Fe, Mn, Mo, Co) and macronutrients (Ca, Mg, K, Na) using absorption and emission (K, Na) atomic spectrometry (AAS, AES) flame technique (PN-ISO 11047). The samples were mineralized using a Start D mineralizer (Millestone) with aqua regia (PN-EN 16,174). Mercury was determined by the Cold Vapour Atomic Absorption Spectrometry (CV-AAS) method using a mercury analyzer (AMA 254, Altec Ltd). The content of sulphates, chlorides, phenol and formaldehyde was determined in water extracts (1:10, S/L) (PN-EN 12,457–1-3); Cl^−^ Mohr titration method (PN-ISO 9297), SO_4_^2−^ by weight method (PN-ISO 9280), phenol and formaldehyde by spectrophotometric methods (PN-76/Z-04045/04; Gadzała-Kopciuch & Buszewski, 2016). Analyses with the use of spectrophotometric methods were performed with the use of UV-1601pc (Shimadzu).

**Fig. 3:**
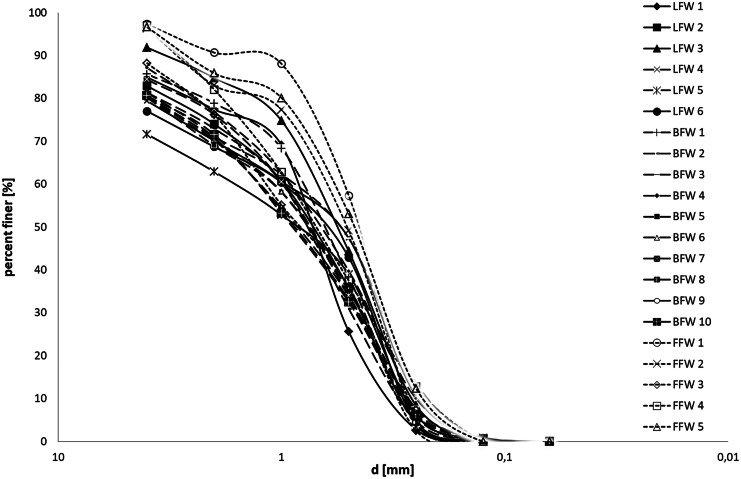
Particle size distribution curves for tested foundry waste. FFW – fresh foundry waste; LFW – landfilled foundry waste; BFW – biologically reclaimed foundry waste

The particle size distribution of foundry waste (4; 2; 1; 0.5; 0.25; 0.125; 0.063 mm) using the weight and aerometric methods of Bouyoucosa, modified by Casagrande and Prószyński, was carried out (Ostrowska et al., 1991) (Fig. 3). Fractional analysis showed that the tested waste consisted mainly of sand fractions (< 2 mm), which was also confirmed by Dayton et al. (2010) and Siddique et al. (2010). The tested waste was characterised by a low percentage of the clay fraction (< 0.2 mm). This fraction affects the binding properties of waste used in construction (Bożym & Klojzy-Karczmarczyk, 2021). Moreover, this fraction may have a positive effect on soil microorganisms by adsorption and immobilization of HM (Yoo et al., 2004). According to Kassim et al. (2005), the grain size distribution of the waste foundry sand is very homogeneous, with approximately 85–95% of the material having a mesh size of 0.6–0.15 mm, and the shape of the grains is usually under an angular or rounded.

### Dehydrogenase (DHA) activity

The INTF method used by other authors investigating DHA in foundry waste was selected for DHA analysis (Dungan et al., 2006; Zhang et al., 2014). Commonly, DHA analysis in Polish soils is performed using the TTC method according to the PN-EN ISO 23753–1 standard (Szara et al., 2020; Uzarowicz et al., 2020). Both methods give divergent results, so they should not be compared. The principle of the INTF method is to reduce the reaction substrate INT (2-p-iodophenyl-3-p-nitrophenyl-5-phenyltetrazolium chloride) to a colored INTF (iodonitrophenylformazan). In the first stage, the moisture content of the sample was determined. The dry mass of the sample was sieved through a sieve with a diameter of 2 mm. 0.50 ± 0.05 g of the sample was weighed into a 20-mL glass test tube. 0.75 mL Tris buffer (1 M, pH 7) and 1 mL of INT solution (5 g·L^−1^) were added. The tube was placed in a water bath (40 °C). Incubation was carried out in the dark for 2 h. After, incubation suspension was filtered. Then, the absorbance of the solutions was measured at *λ* = 464 nm. The result was compared with the calibration curve. The dehydrogenase activity is expressed in μg INTF·g^−1^ DM·2 h^−1^.

### Pot experiment

The aim of the pot experiment was to evaluate changes in the microbial activity (DHA) of the soil mixed with fresh foundry waste (FFW). The effect of ratio and time on changes in DHA activity was assessed. FFW was characterised by no DHA activity. In the pot experiment, soil with high DHA from measurement point no. 20 (arable land) was used. In the experiment, the FFW was mixed with the soil in a mass ratio of 10%, 30% and 50% on a dry basis according to Dungan et al. (2006) and Zhang et al. (2014) experiments.

Air dry soil was mixed with FFW in appropriate mass ratios (10, 30, 50%). In the next step, they were moistened with tap water to humidity of about 15%. A 100 g of the mixture was added to 120-mL pots. Each variant of the experiment was performed in 3 replications. The pots were placed outside, in order to simulate the action of atmospheric factors (solar radiation, air, rainfall). The pots were watered periodically as needed. An experiment was conducted for 6 months in the period May–October 2018. At the same time, a control sample was prepared. Samples were taken every 0, 1, 2, 3 and 6 months from the start of the experiment. In the samples, the dehydrogenase activity was determined.

### Quality control

A quality control of HM in tested samples was carried out with certified reference materials (CRM) “Metals in soil” (SQC001, Merck) and “Rock” (DC73303, NCS). CRM analysis was performed on the basis of the methodology of preparing tested samples of waste and soil. The range of recovery of results for HM in CRM was 85–110%. The control of the analytical range flame atomic absorption spectrometry (FAAS) method was performed with ‘ICP-multielement standard XI’ (Lot: HC394644, Merck, Darmstadt, Germany). The limit of quantification (LOQ) for total metals content was Hg 0.0005 mg kg^−1^ DM; Cd 0.2 mg kg^−1^ DM; Pb, Cu, Zn, Ni, Cr, Mo, Mn, Co 0.5 mg kg^−1^ DM; Fe, Ca, Mg, K, Na 1.0 mg kg^−1^ DM (0.0001% wt). LOQ for other parameters: pH 0.1; LOI 0.1% wt; C_org_ 0.01% wt; N and P 0.01% wt; chloride* and sulphate* 10 mg kg^−1^ DM; phenol* 0.1 mg kg^−1^ DM; formaldehyde* 0.2 mg kg^−1^ DM. The * symbol means leachate.

### Statistical analysis

All experiments and analyses were performed in a minimum of three replications. Mean values, standard deviations (SD) and correlation between DHA and other parameters were calculated using the Statistica v. 13.3 software (TIBCO StatSoft Inc., Poland). Statistical analyses were performed by one-way ANOVA (*t-*test) to determine significant differences between the mean values. Tukey’s HSD (honest significant difference) multiple comparison test (post hoc test) was used to determine homogeneous groups. All analyses were performed at the significance level *α* = 0.05. Homogeneous groups of means are marked in the table with letters a, b, c etc.

### Characteristics of tested waste

Table 2 shows the physicochemical parameters of the tested wastes and soils. The tested wastes were characterised by neutral pH. The pH of the foundry waste is affected by the type of resins and quartz sand used. Quartz sand usually has a slightly acidic pH of about 6 (Dungan et al., 2006). Dayton et al. (2010) report a wide pH range for 39 types of SFS (pH 6.67–10.2). Holtzer et al. (2016) determined a pH of 3.1–4.4 in furan resin foundry waste. Also, dusts contained in casting waste may affect the pH of the waste. In a previous study, foundry dusts from the same foundry had a wide pH range of 5.1–8.2 depending on the place of production in the foundry (Bożym, 2020; Bożym and Klojzy–Karczmarczyk, 2020). Loss on ignition (LOI) is an indicator of the organic matter content of the waste. In foundry waste, organic matter is mainly derived from organic binders. However, in landfilled foundry wastes in the top layer subjected to biological reclamation, organic matter may come from natural decomposition processes (Dungan et al., 2006; Dungan & Dees, 2009; Dungan et al., 2009; Chodak & Niklińska, 2010; Zamulina et al., 2021). FFW characterised the highest LOI and C-org values from tested wastes. The source of organic matter in these wastes is most likely foundry resin residues. On the other hand, in LFW and BFW, organic matter may come from both binder residues and humus. LOI and C-org in these wastes were slightly lower than in local soils. Also, the macronutrient content (N, P) in the foundry wastes was lower than in local soils. Organic binders may be the source of these nutrients, as well as sulfur, in foundry waste (Holtzer et al., 2016). Dayton et al. (2010) determined phosphorus in the tested SFS in wide ranges of 5.41–96.6 mg kg^−1^ DM. The other macronutrient content (Ca, Mg) in the foundry waste was higher than in the local soils, while the Na and K contents were similar. Those metals in foundry waste may come from mineral binders based on sodium or calcium bentonite (Dayton et al., 2010; Dungan & Dees, 2009) or refractory materials (Bożym, 2020). An advantage of the presence of bentonite in foundry waste is the reduction of phenol and heavy metal toxicity to microorganisms (Yoo et al., 2004). Dayton et al. (2010) determined macronutrients in tested 39 types of SFS respectively: Ca 0.094–4.09 g/kg; Mg 0.05–3.20 g/kg; K < 0.05–1.78 g/kg DM.

**Table 2:** Chemical properties of foundry waste and local soils (mean ± SD)

Parameter	LFW (*n* = 6)	BFW (*n* = 10)	FFW (*n* = 5)	LS (*n* = 20)
pH _H2O_	7.67^a^ ± 0.09	7.46^a^ ± 0.13	7.44^a^ ± 0.18	6.50^b^ ± 0.09
LOI [%]	2.12^c^ ± 0.34	1.98^a,b^ ± 0.13	4.09^a^ ± 0.23	3.30^b,c^ ± 0.33
C_org_ [%]	1.04^b^ ± 0.29	1.16^a^ ± 0.12	2.76^a^ ± 0.12	1.30^a^ ± 0.10
N [%]	0.07^a^ ± 0.01	0.05^a^ ± 0.01	0.05^a^ ± 0.01	0.18^b^ ± 0.01
P [%]	0.06^a^ ± 0.01	0.06^a^ ± 0.00	0.03^a^ ± 0.01	0.13^b^ ± 0.01
Cd[mg kg^−1^ DM]	0.59^b^ ± 0.09	0.40^a^ ± 0.06	< 0.2^a,b^	0.58^a^ ± 0.04
Pb [mg kg^−1^ DM]	21.3^b^ ± 1.3	13.5^a^ ± 3.4	36.1^a^ ± 5.3	12.9^a^ ± 1.8
Cu [mg kg^−1^ DM]	22.8^b^ ± 1.2	17.5^a^ ± 1.7	88.2^a^ ± 26.1	9.0^a^ ± 1.1
Zn [mg kg^−1^ DM]	64.3^b^ ± 6.7	58.1^a,b^ ± 3.1	107.1^a^ ± 6.9	52.7^a^ ± 8.3
Ni [mg kg^−1^ DM]	58.3^b,c^ ± 5.4	193.0^a,b^ ± 49.4	129.1^c^ ± 21.2	5.7^a^ ± 1.1
Cr [mg kg^−1^ DM]	30.4^b,c^ ± 4.0	91.2^a,b^ ± 24.3	68.9^c^ ± 12.6	5.0^a^ ± 0.8
Mo [mg kg^−1^ DM]	14.4^c^ ± 4.0	5.6^b^ ± 1.0	25.2^a^ ± 5.2	1.5^a^ ± 0.1
Co [mg kg^−1^ DM]	11.4^a^ ± 2.9	12.4^a^ ± 0.8	15.1^a^ ± 3.4	3.6^b^ ± 0.2
Hg [mg kg^−1^ DM]	0.040^a^ ± 0.002	0.024^a^ ± 0.006	0.027^a^ ± 0.003	0.041^a^ ± 0.004
Fe [% wt]	7.8^c^ ± 1.6	6.3^a^ ± 1.3	14.2^a^ ± 0.5	0.7^b^ ± 0.1
Mn [% wt]	1.56^d^ ± 0.23	0.77^c^ ± 0.24	3.56^b^ ± 0.20	0.02^a^ ± 0.00
Mg [% wt]	1.38^a,b^ ± 0.33	0.59^b^ ± 0.18	1.32^a^ ± 0.40	0.05^c^ ± 0.01
Ca [% wt]	5.23^a^ ± 0.68	2.39^a^ ± 0.82	6.15^c^ ± 1.02	0.05^b^ ± 0.01
Na [% wt]	0.04^a^ ± 0.00	0.04^a^ ± 0.00	0.05^a^ ± 0.00	0.06^a^ ± 0.01
K [% wt]	0.08^b^ ± 0.03	0.05^a^ ± 0.00	0.16^a^ ± 0.03	0.05^a^ ± 0.00
Chlorides [mg kg^−1^ DM]*	299^b^ ± 41	173^b^ ± 22	381^a^ ± 87	164^a^ ± 11
Sulphur [mg kg^−1^ DM]*	177^c^ ± 26	149^a^ ± 17	359^a^ ± 86	25^b^ ± 1
Phenol [mg kg^−1^ DM]*	1.4^b^ ± 0.5	0.6^a^ ± 0.1	2.8^c^ ± 0.9	< 0.1^a^
Formaldehyde [mg kg^−1^ DM]*	29.2^b^ ± 2.8	1.5^a^ ± 0.2	49.5^c^ ± 4.2	< 0.2^a^

*FFW* Fresh Foundry Waste *LFW* Landfilled Foundry Waste, *BFW* Biologically Reclaimed Foundry Waste, *LS* Local Soil, *LOI* Loss of Ignition

^*^In leachates [S/L, 1/10] according to PN-EN 12,457

Data with the same letters show nonsignificant differences, while different letters show significant difference at *p* < 0.05 (HSD Tukey’s test)

**Fig. 4:**
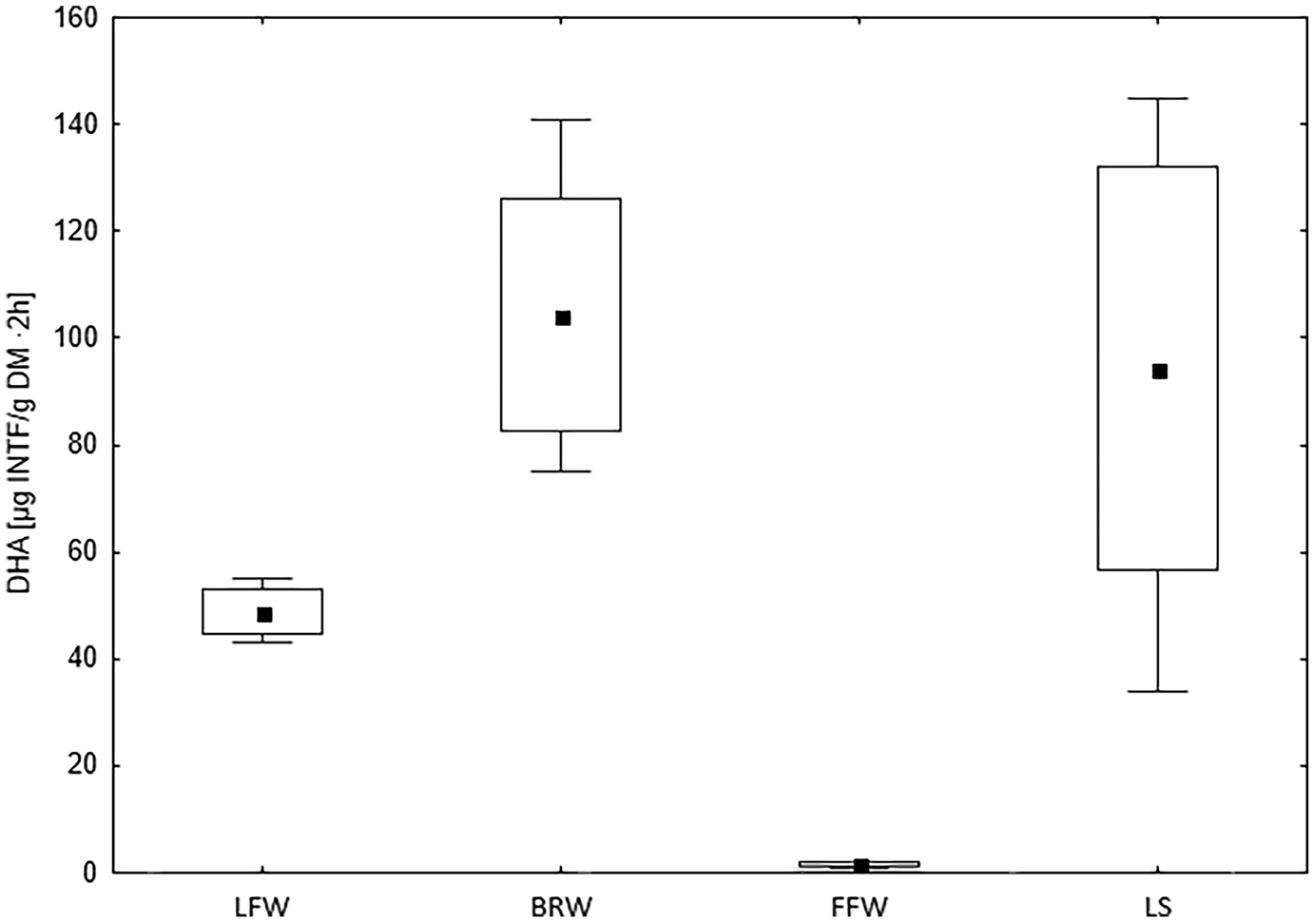
Dehydrogenase activity of waste and soil samples. LFW – landfilled foundry waste; BFW – biologically reclaimed foundry waste; FFW – fresh foundry waste; LS – local soil

The heavy metal content, except mercury, in the tested foundry waste was higher than in local soils, but the content was within the limits given by other authors (Dayton et al., 2010; Dungan & Dees, 2009). Most metals in foundry waste are found in immobile forms, but landfilling may increase the percentage of bioavailable forms (Bożym, 2022). This is important for the use of these wastes in agrotechnology. The high content of iron and manganese in the tested foundry wastes is due to the presence of cast metal residues in the wastes. Dungan and Dees (2009) and Dayton et al. (2010) also found a high percentage of Fe and Mn in foundry waste. Sulphate and chloride leaching is an indicator of waste salinity of waste. When foundry waste is landfilled and used in agrotechnology, this parameter may contribute to environmental pollution. Besides, salinity affects the reduction of soil microbial activity (Filipovic et al., 2020; Shi et al., 2019). The leachability of chloride and sulphate from the tested wastes was higher than in soils. Especially, high leaching was manifested by FFW. These wastes also had the highest leachability of phenol and formaldehyde. This is due to the presence of residues of phenol–formaldehyde resin used in the foundry. In the other wastes, the leachability of these organic pollutants was lower. Some authors suggest the use of organic binders from foundry waste by soil microorganisms as a carbon source (Dungan et al., 2006; Dungan & Dees, 2009; Dungan et al., 2009; Watson et al., 2017). The microbial activity of the tested foundry waste, measured as DHA, varied within a wide range (Fig. 4). Contents at the limit of quantification were found for FFW, which is a predictable effect since these wastes were not influenced by soil microorganisms. A pot experiment was conducted based on these wastes to evaluate the microbial activity of soils mixed with these wastes in different proportions. The highest DHA value was obtained for BFW (mean 104.2 ± 21.7 µg INTF·g^−1^ DM·2 h^−1^), which is the waste subjected to biological reclamation. It is known that plant cover and soil microbial activity affect the microbial activity of landfilled waste (Chodak & Niklińska, 2010; Zamulina et al., 2021). The DHA value determined for this waste was higher than the average for local soils. A possible cause of this effect is the high content of soluble organic matter in the BFW used by microorganisms as a source of carbon and energy (Dungan et al., 2006, 2009; Singh & Kumar, 2019). Tested wastes, which were landfilled since the 1990s (LFW), characterised a lower than BFW biological activity (mean 48.8 ± 4.2 µg INTF·g^−1^ DM·2 h^−1^) than BFW.

It is known that HM content, salinity and organic pollutants may affect DHA values (Filipovic et al., 2020; Tiwari et al., 2019). Correlation analysis between waste parameters and microbial activity was performed. Based on the results obtained, several statistically significant correlations were found. In foundry waste, a positive correlation was found between N/DHA (*r* = 0.814) and Hg/DHA (*r* = 0.909), which is an unexpected result. The mercury content of the tested waste was low, at levels similar to soils. Such a low content probably did not have a toxic effect on microorganisms, and had a stimulatory effect in this study. The stimulatory effect of some metals in soil is confirmed by Peng et al. (2009) and Tiwari et al. (2019). Excess metal levels may have an inhibitory effect on DHA. This is confirmed by the correlation results; negative correlation for Cr/DHA (*r* = − 0.856), Ni/DHA (*r* = − 0.711) and for phenol/DHA (*r* = − 0.824) and formaldehyde/DHA (*r* = − 0.940) was found. The content of phenol and formaldehyde in the tested wastes was higher than in the soils and could have an inhibitory effect on DHA.

### Characteristics of the tested soils

In the studied area, one may distinguish soils formed as a result of autogenic processes (brown earth soils), semi-hydrogenic (black earth soils), hydrogenic (marshy and post-marsh soils), inflow (alluvial soils) and anthropogenic processes (industrial soils). In typological terms, black earths, alluvial soils and brown earth soils predominate, with a smaller share of luvisols (pseudo-podzolic), peaty and silty soils and a trace of peaty soils (Spatial Planning, 2014). The tested soil samples were classified in previous studies as light soils, with sand and loamy sand predominating (Bożym & Klojzy–Karczmarczyk, 2021). Soil samples were collected from cultivated fields, allotment gardens, meadows, wastelands, forests and urban areas. According to the Ordinance (Journal of Laws, 2016, item 1395), the tested soils belonging to all groups (I-IV) did not show contamination with HM (Cr, Zn, Cd, Co, Cu, Mo, Ni, Pb, Hg) and phenol. According to the classification of guidelines of the Polish Institute of Soil Science and Plant Cultivation (IUNG Puławy, 2017), the tested soils are classified as light soils with neutral pH and natural trace metal content. According to the guidelines, these soils can be used for all horticultural and agricultural crops, in accordance with the principles of rational use of agricultural production space. The tested soils were moderately rich in macronutrients and organic carbon. Additionally, formaldehyde, sulphate and chloride contents were determined in the soil extracts. However, these parameters are not standardized in Poland. The analyses were performed for comparison with the tested wastes.

DHA analysis showed a wide range of microbial activity in the tested soils. The lowest activity was found in meadow, forest (55–63 µg INTF·g^−1^ DM·2 h^−1^) and wasteland soils (34–55 µg µg INTF·g^−1^ DM·2 h^−1^). High DHA values were determined in allotment garden soils (120–145 µg INTF·g^−1^ DM·2 h^−1^). Soils from cultivated fields were characterised by a wide range of DHA (45–125 µg INTF·g^−1^ DM·2 h^−1^). The mean DHA for the tested soils was 94.3 µg INTF·g^−1^ DM·2 h^−1^ (Fig. 4). In the current study, there was no statistically significant correlation between DHA in soil and other parameters (*r* < 0.6; *p* < 0.05). Hanajík et al. (2017) determined a higher value of DHA in forest soils at 120–250 µg INTF·g^−1^ DM·48 h^−1^. The authors conducted their study after 48 h, not after 2 h as in the used methodology, which could have influenced the higher result. The authors also found a positive correlation between microbial biomass and DHA. Hamidović et al. (2020) found the highest DHA for forest soils and arable land, contaminated with metals compared to wasteland and post-mining land (overburden). Some authors also stated higher microbial activity in arable land compared to wasteland, probably due to the increased amount of nutrients (Chodak & Niklińska, 2010; Shi et al., 2020; Trivedi et al., 2016). The decrease in biomass and microbial activity is influenced by low organic matter content and reduced nutrient supply (Ciarkowska & Gambus, 2020). Ramirez et al. (2021) investigated the effect of soil fertilisation with sewage sludge contaminated with petroleum compounds with the addition of surfactants. The authors concluded that the non-fertilised soil showed the highest DHA value of over 200 µg INTF·g^−1^ DM·2 h^−1^, while the addition of sewage sludge contaminated with surfactants to the soil reduced the DHA to at least 100 µg INTF·g^−1^ DM·2 h^−1^, and to even below 50 µg INTF·g^−1^ DM·2 h^−1^. Biologically reclaimed industrial sites characterise higher DHA activity due to the influence of vegetation cover than on non-reclaimed sites (Chodak & Niklińska, 2010; Howard, 2017). Under conditions of thin vegetative cover, the content of soluble organic matter available to microorganisms may become a limiting factor for microbial abundance and activity (Zamulina et al., 2021). El Gouzi et al. (2015) investigated DHA as an index of phenylurea herbicide degradation in agricultural calcareous soil (Spain) and organic forest soil (Morocco). The authors found higher DHA for calcareous soil than for forest soil, probably due to higher nutrient availability and the content of dissolved organic carbon. However, this higher enzymatic activity did not have an impact to faster degradation of the herbicides, probably because the microorganisms preferred a non-herbicide-derived carbon source. The studies of various authors indicate that DHA is influenced by dissolved organic carbon (Brkljaca et al., 2019; Howard, 2017; Zamulina et al., 2021). The source of carbon may be organic pollutants such as phenols, furfuryl or urea derivatives (El Gouzi et al., 2015; Singh & Kumar, 2019), which also contain foundry waste, stimulating the activity of microorganisms (Dungan et al., 2006, 2009; Zhang et al., 2014). In the current study, soil from arable fields with the highest DHA activity (125 µg INTF·g^−1^ DM·2 h^−1^; sample no. 20) was used for the pot experiment.

### The pot experiment

**Fig. 5:**
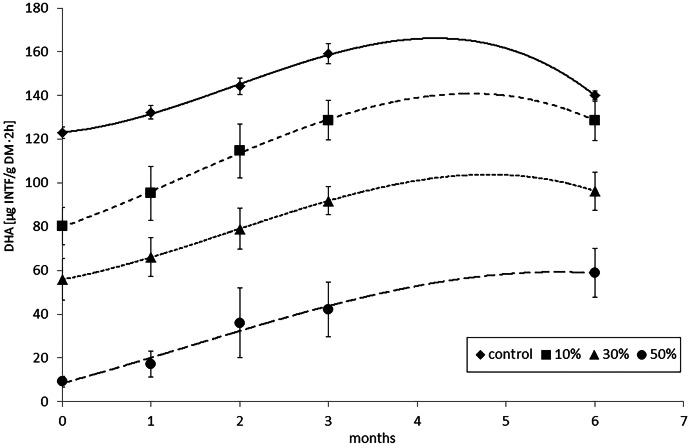
DHA activity in soil samples (control) and blended soil and FFW with 10%, 30%, and 50% ratio after 0, 1, 2, 3 and 6 months of treatment

Figure 5 shows the changes in DHA over time. Values were averaged for five sample types. Untreated soil (control) showed the highest microbial activity. Increasing the percentage of FFW resulted in lower DHA, probably due to dilution of the substrate, which consequently reduces the total microbial population in treated soil and thus results in reduced DHA (Dungan et al., 2006; Zhang et al., 2014). A possible cause of DHA reduction could also be the presence metals, core binders and other factors of SFS (Dungan et al., 2006). In the first 3 months, an increase in DHA values was recorded in all variants of the experiment. This increase in DHA may be the result of the activity of microorganisms using residual organic binders as a carbon source. An increase in DHA for SFS-treated soil containing organic binders compared to mineral binders was also observed by Dungan et al. (2006). These authors also noted that the DHA of soils treated with SFS from iron foundries is affected by the presence of Fe_2_O_3_, but the mechanism of this effect is unknown. In the current study, DHA values were found to remain constant for the 30% and 50% FFW variants after 6 months of the experiment. However, for the 10% variant, a slight increase in the value was recorded, which may indicate favorable conditions for the development of soil microorganisms and non-exhaustion of nutrients. On the other hand, in untreated soil (control), a decrease in DHA was observed after 6 months. The reason could be the depletion of organic matter in the soil needed as a carbon source for microorganisms. In addition, temperature differences occurring between May and October may have influenced the results of the experiment. The temperature may be an important factor which influences HA. These experiments were conducted outdoors, not under controlled conditions in a laboratory. Future experiments should be designed to assess the effects of temperature and sunlight on the DHA of the SFS-treated soil. These aspects were not investigated by Dungan et al. (2006) and Zhang et al. (2014). After 6 months of the experiment, differences between control and treated soil were found to decrease. A possible cause of this effect was the similar growth of microbial colonies and their activity in all variants (Dungan et al., 2006; Zhang et al., 2014). The smallest differences occurred in the soil with 10% FFW, from 35% at the beginning of the experiment to 8% after 6 months. In this case, a possible cause could be the loss of organic matter in the substrate. Despite the beneficial microbial effects in SFS soils containing organic binders, it is not possible to use them for artificial substrates in the USA (Dayton et al., 2010; EPA Report, 2014). Dungan et al. (2006) suggest that the maximum addition of foundry waste to soils should not exceed 30% by weight, as higher percentages may cause undesirable effects of clumping and high water retention. In contrast, Zhang et al. (2014) suggest that this ratio should be lowered to 15% by weight. It seems that the most optimal dose of the tested SFS was 10% wt, characterised by the highest DHA values, similar to the untreated soil. In the other variants (30% and 50% wt), the increase in DHA was also significant. However, the DHA value was lower than in the control, even after 6 months of the experiment. The degree of degradation of organic binder residues was not assessed in the current studies. These analyses will be carried out in the next experiments, taking into account the influence of atmospheric factors.

**Fig. 6:**
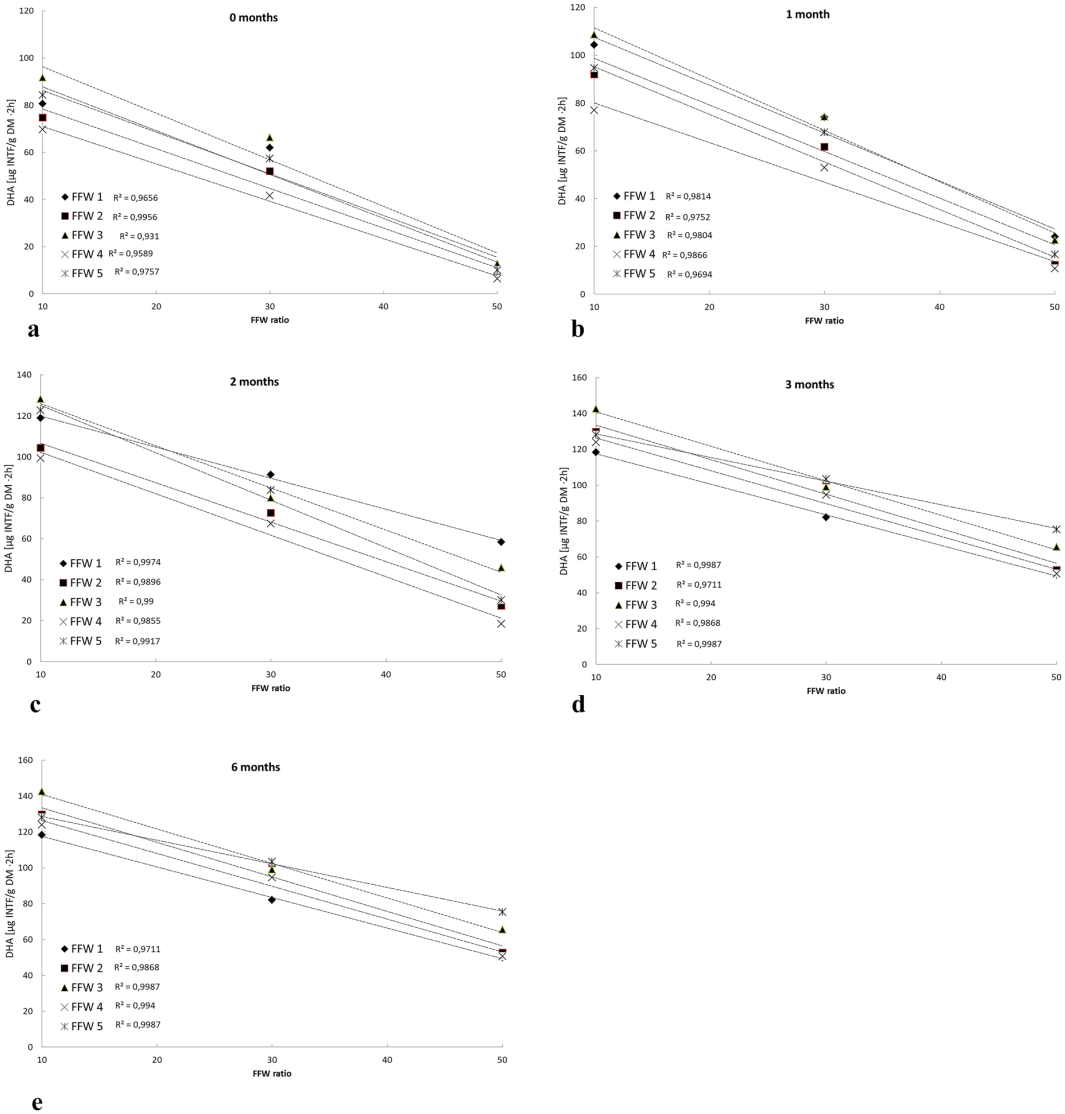
Correlation between the dehydrogenase activity of FFW blending ratio after 0 (**a**), 1 (**b**), 2 (**c**), 3 (**d**) and 6 (**e**) months of treatment in pot experiment

The correlation coefficients (*R*^2^) calculated for the correlation of DHA and FFW blending ratio during 0, 1, 2, 3 and 6 months of the experiment (Fig. 6) showed that as the percentage of FFW increased, the value of DHA decreased, especially at the beginning of the experiment. As mentioned above, this is probably the effect of soil dilution by the FFW addition, which did not show any microbial activity. Zhang et al. (2014) in their study confirm the effect of soil dilution by foundry waste on the reduction of DHA from about 100 INTF·g^−1^ DM·2 h^−1^ (10% wt SFS) to about 50–80 INTF·g^−1^ DM·2 h^−1^ (30% wt SFS) and 10–60 INTF·g^−1^ DM·2 h^−1^ (40% et. SFS). The authors found a decrease in DHA after 12 weeks of the experiment as a result of a reduction in the content of organic carbon and nutrients. In the current study, there was no relationship between the duration of the experiment and the correlation coefficient value. In contrast, Zhang et al. (2014) found an increase in the correlation coefficient over time; the highest coefficients were achieved by the authors after 12 weeks of the pot experiment.

## Conclusions

Microbial activity depended on the type of landfilled foundry waste. BFW showed the highest activity, similar to local soils. FLW showed moderate microbial activity. In contrast, fresh foundry waste did not show this activity. There was a negative correlation between Cr, Ni, phenol and formaldehyde and DHA in the waste.

Local soils were not contaminated with HM and had different biological activities. Soils from allotment gardens showed the highest activity. Soils from cultivated fields were characterised by a wide range of DHA. Soils from wasteland and forest showed the lowest activity. There was no effect of HM and other soil constituents on DHA.

The pot experiment showed that increasing the percentage of foundry waste in soil negatively affected DHA, probably due to soil dilution rather than contaminant action. DHA was found to increase in subsequent months of the pot experiment for all variants except the control. In untreated soil, a decrease in DHA was found after 6 months of the experiment, probably due to nutrient depletion. The greatest increase in microbial activity was found for the 10% wt FFW variant, which was most similar to the untreated soil. In this experiment, the optimum percentage of foundry waste from the point of view of microbial activity was 10% wt, although some authors suggested a percentage of 15% wt or even 30% wt.

## Data Availability

The datasets generated during and/or analysed during the current study are available from the corresponding author on reasonable request.
